# Resistin as a Prooxidant Factor and Predictor of Endothelium Damage in Patients with Mild Acute Pancreatitis Exposed to Tobacco Smoke Xenobiotics

**DOI:** 10.1155/2017/3039765

**Published:** 2017-09-26

**Authors:** Milena Ściskalska, Grzegorz Marek, Zygmunt Grzebieniak, Halina Milnerowicz

**Affiliations:** ^1^Department of Biomedical and Environmental Analysis, Faculty of Pharmacy, Wroclaw Medical University, Wroclaw, Poland; ^2^Second Department of General and Oncological Surgery, Wroclaw Medical University, Wroclaw, Poland

## Abstract

**Objectives:**

The study was aimed to assess the influence of tobacco smoke exposure on the intensity of inflammation measured by IL-6, *α*_1_-antitripsin (AAT) and *α*_1_-acid glycoprotein (AGP) concentrations, and Cd level and oxidative stress intensity measured by advanced oxidation protein product (AOPP) concentration in the blood of healthy subjects and AP patients during hospitalization. Endothelin-1 (ET-1) and resistin concentrations, markers of endothelium injury, were determined.

**Results:**

An increased IL-6 concentration in healthy smokers compared to nonsmokers and AP patients compared to controls was shown. An increased AAT and AGP concentrations during hospitalization of AP patients were noted, in both smokers (AAT, AGP) and nonsmokers (AAT). In comparison to control groups, in AP patients, a 2-fold increased resistin concentration correlating with ET-1 concentration and decreased albumin concentration accompanied by increased AOPP concentration were demonstrated. AOPP concentration was higher in smokers with AP compared to nonsmokers and gradually enhanced during their hospitalization.

**Conclusions:**

Tobacco smoke exposure can have a proinflammatory effect in both healthy subjects and AP patients. Increased resistin concentration in AP patients negatively correlating with albumin concentration has prooxidative effect on this protein resulting in enhanced AOPP level. Increased resistin concentration can intensify AAT and AGP production during AP.

## 1. Introduction

Tobacco smoke xenobiotics contribute to indirectly generation of free radicals and oxidative stress into organism [1, 2]. Free radicals and Cd included in the smoke are responsible for initiation of proinflammatory cytokine expression, which can additionally intensify a local inflammation [3–5]. There are some studies which evidenced that smoke xenobiotics can have a toxic effect on human organs, especially on the pancreatic tissue [6–8]. The most toxicity against pancreas showed nicotine and its metabolites, especially cotinine [9]. This substance is able to activate cell surface receptors on the exocrine pancreas, including nicotinic acetylcholine receptor, and mediate pancreatitis [9]. Therefore, smoking is considered as a major factor inducing acute pancreatitis (AP) [10].

A clinical course of AP is dependent on the overlapping metabolic changes associated with proinflammatory cytokine action including interleukin-6 (IL-6). IL-6 is released by macrophages in response to tissue damage. It appears in the blood 24–36 hours earlier than C-reactive protein, commonly used as an indicator of inflammation. IL-6 can be a marker of complication in the course of AP [11]. It has a proinflammatory effect by modulation of the hepatic expression of acute-phase proteins, among others *α*_1_-antitripsin (AAT) and *α*_1_-acid glycoprotein (AGP) [11–14].

AAT and AGP are glycoproteins considered as anti-inflammatory agents [12, 13]. AAT is expressed in monocytes, macrophages, neutrophils, endothelial cells, and pancreatic islet *α* and *β* cells [12]. AAT appears to effectively interfere with inflammatory process by decreasing the production of proinflammatory cytokines and protease inhibition [12]. However, an increased AGP level in the blood is associated with systemic tissue injury and exposure to tobacco smoke and cytokines [15]. Biological role of AGP is unclear [13, 15], but it is known that this protein is required to maintain capillary permeability probably by increasing the polyanionic charge selectivity of the endothelial barrier, which can indicate that AGP is produced by endothelial cells [13]. AGP is also a potent inhibitor of neutrophil chemotaxis and oxidative metabolism [13].

Oxidative stress in the blood can cause harmful effects on vascular endothelium [16]. Damaged endothelial cells can intensify the release of endothelin-1 (ET-1), a major vasoconstrictive factor, which is also produced in the pancreatic tissue [2, 17]. It was shown that ET-1 may play a role in the pathogenesis of pancreatitis by a decrease in pancreatic perfusion and contribute to oxidative stress [17]. ET-1 also exerts a proinflammatory effect by stimulation of IL-6 synthesis in endothelial cells causing intensification of existing inflammation [2, 17]. Dysfunction of endothelium is also related with high concentration of resistin, 12.5 kDa cysteine-rich peptide, expressed primary by adipocytes and mononuclear leukocytes, macrophages, and spleen and bone marrow cells [18–20]. Resistin can act as prooxidant mediator for endothelial dysfunction [19]. Additionally, this protein seems to be involved in the requirement of immune cells and secretion of inflammatory cytokines [18–20]. It was shown that this peptide may represent a new effective indicator to predict the severity of AP [20].

Endothelium damage and oxidative stress induced by smoke xenobiotics seem to be related with acute pancreatitis [10, 21]. One of the markers used to assess oxidative stress is advanced oxidation protein products (AOPP) [22]. AOPP is formed mainly from oxidized albumin—a predominant antioxidant in plasma [23]. In this study, the influence of tobacco smoke exposure on the intensity of inflammation measured by IL-6, AAT and AGP concentrations, and Cd level and the intensity of oxidative stress measured by AOPP concentration in the blood of healthy subjects and the patients with mild AP during hospitalization was monitored. The study also aimed to assess the concentration of ET-1 and resistin as markers of endothelium injury in this population.

## 2. Material and Methods

### 2.1. Materials

The study group consisted of the patients hospitalized in the Second Department of General and Oncological Surgery, Wroclaw Medical University and healthy volunteers classified as a control group. The subjects were included into the study in years 2014–2016. The study protocol was approved by the Local Bioethics Committee of Wroclaw University of Medicine (number: KB-592/2013). The criteria for the inclusion of patients to the study were shown in Table 1.

The volunteers were classified as a control group based on the research conducted by clinicians of primary medical care. We excluded from the study the individuals with diagnosed disease as well as alcohol and drug abusers. To confirm the lack of alcohol abusers in the examined subjects, the carbohydrate-deficient transferrin (CDT) (CEofix CDT kit for Beckman Coulter P/ACE MDQ Series; ref. number: 844111036), a biomarker for long-term alcohol consumption, was estimated.

All hospitalized patients and healthy volunteers had been informed about the aim of the study and gave their consent. A personal interview about their lifestyle was carried out. Participants were asked about their health and nutritional habits, anthropometry, the use of dietary supplements/medications, frequency of alcohol intake, and smoking history. The alcohol intake was expressed in grams of ethanol per day. Smoking status was categorized as never and current and verified by determination of serum cotinine concentrations, a metabolite of nicotine. Samples of patients and control groups were divided into two subgroups: smokers (cotinine concentration > 25 ng/ml) and nonsmokers (cotinine concentration < 25 ng/ml); the intensity of smoking was evaluated in pack years, defined as the number of smoked cigarettes per day multiplied by the number of years of smoking divided by 20 (20 cigarettes/pack). Body mass index (BMI) was calculated as weight (kg)/(height)^2^ (m). Data about age, BMI, Ranson scale, smoking habits, and alcohol exposure of volunteers and patients with AP in Table 2 were presented.

### 2.2. Sample Preparation

The investigations were performed in serum, plasma, and erythrocyte lysate derived from 35 patients (7 women and 4 men, who were nonsmokers, and 7 women and 17 men, who were smokers) and 95 healthy volunteers (52 women and 20 men, who were non-smokers, and 15 women and 8 men, who were smokers). Venous blood was collected in the morning, after 12 h fasting. The blood samples were collected on the patients' admission to the hospital and on the 3rd and 7th day of hospitalization. Serum was obtained according to the standard procedure by taking venous blood for disposable trace element-free tubes (ref. number: 368815, Becton Dickinson, Germany) with serum-clotting activator, left at 25°C to complete thrombosis, and centrifuged (1200*g*/20 min). In order to obtain plasma and erythrocyte lysate, whole blood was drawn into tubes containing heparin (ref. number: 368886, Becton Dickinson, Germany) and EDTA with aprotinin (50 KIU per ml of blood) (ref. number: 361017, Becton Dickinson, Germany) for ET-1 determination. The whole blood was centrifuged (2500*g*/15 min) to separate the plasma and buffy coat from erythrocyte pellet. The erythrocytes were then directly transferred to other tubes to prevent hemolysis. The erythrocyte pellet was washed in an equal volume of ice-cold 0.9% NaCl. This process was repeated twice. The washed cells were lysed by the addition of ice-cold double distilled water (1 : 1.4). The resulting lysate was used for the assays. The obtained plasma and erythrocyte lysate were portioned and stored in sealed tubes (ref. number: 0030102.002, Eppendorf, Germany). The samples were stored at −80°C until analysis.

### 2.3. Methods

Cotinine concentration was measured using the commercial cotinine ELISA test (ref. number: EIA-3242, DRG International Inc., USA). It provides qualitative screening results for cotinine in human serum at a cut-off concentration of 25 ng/ml.

Cd concentration in erythrocyte lysate was determined by graphite furnace atomic absorption spectrometry (GFAAS) using SOLAAR M6, Thermo Elemental Co. The absorbance was measured at *λ* = 228.8 nm, with Zeeman background correction. Reference materials (Recipe, BCR) were used to determine the calibration curve and controls.

Interleukin-6 (IL-6) determination in plasma was performed with human IL-6 DuoSet ELISA test (ref. number: DY206-05, R&D Systems, USA). *α*1-Antitripsin (AAT) concentration in serum was measured by ELISA method using alpha-1-antitripsin clearance ELISA (cat. number: K6752, Immundiagnostic, Germany). *α*1-Acid glycoprotein (AGP) concentration was determined in serum using AssayMax Human Alpha-1-Acid Glycoprotein ELISA Kit (cat. number: EG5001-1, Assaypro, USA). Resistin concentration in plasma was measured with Quantikine ELISA Human Resistin test (cat. number: DRSN00, R&D Systems Inc., USA and Canada) and plasma endothelin-1 (ET-1) concentration with endothelin (1-21) test (cat. number: BI-20052, Biomedica, Austria).

Albumin concentration in plasma was determined by using the bromocresol purple (BCP) (cat. number 115-40-2, Sigma-Aldrich, Germany), which reacted with albumin in phosphate buffer saline (PBS) at pH = 6.8. The concentration of albumin standard (cat. number 70024-90-70, Sigma-Aldrich) was determined using the molar absorption coefficient (*A*_280 nm_ 0.1% = 0.5). The determination was performed as described earlier [24]. Absorbance of the standards and of the samples was measured at *λ* = 603 nm.

Determination of AOPP concentration in plasma was performed by spectrophotometric method developed by Witko-Sarsat et al. [25], based on the reaction of AOPP with potassium iodide in acidic conditions as described earlier [24]. The absorbance of samples was measured at *λ* = 340 nm. The results of determination were expressed in micromoles per liter chloramine T as equivalents, converted per gram of albumin, and expressed as AOPP/albumin ratio (*μ*mol/g).

### 2.4. Statistical Analysis

The data is expressed as median (range) values. The differences between the examined groups were tested using the Student's *t*-test or a nonparametric Mann–Whitney *U* test. The normality of the variables was analyzed using the Shapiro-Wilk *W* test. In order to verify correlations between examined parameters, the multiple linear regression models were performed. In all instances, *p* < 0.05 was considered statistically significant. Statistical calculations were done using the Statistica Software Package, version 10.0 (Polish version: StatSoft, Krakow, Poland).

## 3. Results

### 3.1. The Effect of Tobacco Smoke Exposure on the Concentrations of Cotinine, Cd and IL-6, AAT, and AGP in the Blood of the Control Group and the Patients with AP

Higher cotinine levels in the blood of smokers (compared to nonsmokers in both control group (*p* < 0.0001) and the patients with AP (*p* < 0.0001, *p* = 0.0006, and *p* = 0.0108 for the 1st, 3rd, and 7th day of hospitalization, resp.)) were demonstrated. In the groups of smoking patients with AP, in whom the intensity of smoking was assessed on 15 pack-years, a near 2-fold higher concentration of cotinine compared to smokers of control groups (with intensity of smoking about 3 pack-years) was shown (*p* < 0.0001) (Table 3).

Increased Cd concentrations in the blood of smokers compared to nonsmokers, in both healthy subjects (*p* = 0.0001) and the patients with AP (*p* < 0.0001, *p* = 0.0052, and *p* = 0.0491 for the 1st, 2nd, and 3rd day of hospitalization, resp.), was shown. Additionally, it was noted that Cd concentration in the patients with AP increased more than 6-fold in the group of nonsmokers (*p* < 0.0001) and more than 4-fold higher in the group of smokers (*p* = 0.0005) when compared to appropriate control groups (Table 3).

IL-6 concentration was higher in the plasma of smoking healthy subjects compared to nonsmokers of this group (*p* = 0.0413). An increased IL-6 concentration in the blood of nonsmoking and smoking patients with AP, when compared to appropriate control groups (*p* < 0.0001 and *p* = 0.0193 for nonsmokers and smokers, resp.), was demonstrated (Table 3).

AAT concentration was 50% higher in the blood of nonsmoking patients with AP compared to healthy nonsmokers (*p* = 0.0473). It was observed that AAT concentration was gradually elevated during hospitalization. The concentrations of this protein in the blood of nonsmoking AP patients increased about 2-fold in the 7th day of hospitalization compared to the admission (*p* = 0.0275) and the 3rd day of hospitalization (*p* = 0.0497). In the blood of smoking AP patients, AAT concentration was by 80% higher in the 7th day of hospitalization when compared to the 1st (*p* = 0.0017) and 3rd day of hospitalization (*p* = 0.0491) (Table 3).

AGP concentration was nearly 2-fold higher in the blood of patients with AP, in both nonsmokers (*p* = 0.0136) and smokers (*p* < 0.0001) compared to control groups. Additionally, the changes in AGP concentration during hospitalization of smokers were demonstrated. A 60% increase in the concentration of this protein in the 7th day of hospitalization compared to admission (*p* = 0.0010) and the 3rd day of hospitalization (*p* = 0.0400) in the blood of smoking AP patients was shown (Table 3).

### 3.2. The Effect of Tobacco Smoke Exposure on the Concentrations of Albumin, AOPP, and the Value of AOPP/Albumin Ratio in the Blood of Control Group and the Patients with AP

More than 40% decrease in albumin concentration in plasma of patients with AP compared to appropriate control groups, in both non-smokers (*p* < 0.0001) and smokers (*p* < 0.0001), was shown. Additionally, a decreased concentration of this parameter in plasma of smoking patients with AP during hospitalization was noted. About 20% decrease in albumin concentration in the 3rd and 7th day of hospitalization compared to the concentration of these parameters on admission (*p* = 0.0041 and *p* = 0.0114, resp.) was shown (Table 4).

AOPP concentration in plasma of patients with AP was increased by 34% in nonsmokers (*p* = 0.0012) and more than 80% in smokers (*p* = 0.0004) compared to appropriate control groups. It was also shown that the AOPP concentration in the blood of smoking patients with AP gradually increased during hospitalization. It was demonstrated that there were about 30% increase in AOPP concentration in the 7th day of hospitalization compared to the 1st day of hospitalization (*p* = 0.0115) and more than 10% increase compared to the 3rd day of hospitalization (*p* = 0.0294) (Table 4).

AOPP/albumin ratio was increased more than 2-fold in the blood of patients with AP compared to control groups (*p* < 0.0001 in both nonsmoking and smoking group), and it was gradually increased during hospitalization of smoking patients with AP. The value of AOPP/albumin ratio was increased by about 30% in the 3rd day of hospitalization (*p* = 0.0249) and 40% in 7th day of hospitalization (*p* = 0.0072) compared to AOPP/albumin ratio in the 1st day of hospitalization (Table 4).

### 3.3. The Effect of Tobacco Smoke Exposure on the Concentrations of Resistin and ET-1 in the Blood of the Control Group and the Patients with AP

A 2-fold increase in resistin concentration in the blood of patients with AP compared to control groups, in both smokers (*p* = 0.0001) and nonsmokers (*p* = 0.0014), was demonstrated. More than 2-fold increase in the concentration of this parameter in the blood of nonsmoking patients with AP in the 7th day of hospitalization compared to the admission (*p* = 0.0242) (Table 4) was shown.

It was shown that there were no differences in ET-1 concentrations between examined groups. However, it was noted that there was a near 2-fold higher range in the groups of smokers (both healthy subjects and patients with AP) when compared to nonsmoking groups (Table 4).

The correlations between examined parameters were performed. The correlation coefficients between parameters determined in the blood of control group and patients with AP in Table 5 were presented.

## 4. Discussion

Compounds of tobacco smoke and free radicals can have a harmful effect on vascular endothelium causing its damage by initiation of inflammation and oxidative stress [26]. Injured endothelial cells may secrete vasoconstriction factors and reduce pancreatic microcirculation, which play a crucial role in pathogenesis of AP [27].

In our study, we examined the patients with AP in ages 26–76, who were more exposed to tobacco smoke xenobiotics than smokers of the control group as indicated by elevated cotinine concentration and 4-fold increase in Cd level. A 6-fold increase in Cd concentration observed in nonsmoking patients with AP compared to healthy nonsmokers can be a result of age-dependent Cd accumulation in organism from environment as other studies have reported [28, 29]. Tobacco smoke components have the ability to induce a systemic inflammation and secretion of proinflammatory cytokines [1, 30]. Śliwińska-Mossoń et al. [30] shown that IL-6 concentration in the blood of smokers was increased compared to nonsmokers [30]. It is consistent with presented study, in which an increase in IL-6 concentration correlated with cotinine level in the group of smoking AP patients was shown, which evidenced a proinflammatory effect of tobacco smoke xenobiotics.

In the presented study, molecular changes leading to inflammatory cascade activation were already shown in young smokers. It can confirm an increase in IL-6 concentration corresponding with a little elevation of AGP concentration as acute phase protein. A 2-fold increase in the concentration of determined acute phase proteins (AAT and AGP) in the blood of AP patients was also demonstrated. It can indicate that both AAT and AGP appeared in the blood in response to tissue injury in the course of AP. In other studies, it was also reported that an increase in IL-6 concentration as a result of inflammation can induce the expression of acute phase proteins [12, 13, 15]. Additionally, in other researches, it was shown that elevated concentration of AAT as a protease inhibitor is related with anti-inflammatory effect [12]. AAT in return can block protease-activated receptors involved in IL-6 release from cells [12]. Therefore, it was observed in our study that no changes in IL-6 concentration during hospitalization of AP patients (both smokers and nonsmokers) accompanied by near 2-fold increases in AAT levels can suggest that this protein is involved in the blanking of inflammatory process induced during AP and/or tobacco smoke exposure. Additionally, we confirmed that smoke xenobiotics can influence on increased AGP concentration correlating with Cd level in the group of smoking AP patients, which is consistent with other studies [15]. In our study, it was shown that the concentration of this protein was gradually elevated during hospitalization of smokers in contrast to nonsmokers. It can be caused by the exposure to tobacco smoke xenobiotics, which may be a major modulator of AGP gene expression mediated by NF-*κ*B, which in other studies were reported [1, 13].

The interaction of inflammation and environmental stressors such as tobacco smoke xenobiotics leads to the activation of oxidative inflammatory cascade [31]. It can result in the progression of inflammation and the formation of oxidative stress markers. In the studies conducted by other researches, an enhanced level of oxidized protein in the course AP was shown [32, 33]. Additionally, it was demonstrated that the content of carbonyl groups in proteins, which are included in large amounts in the structure of AOPP, was exacerbated with AP progress [33]. In our study, AOPP concentration as a marker of oxidative stress and AOPP/albumin ratio were increased during hospitalization of smoking patients with AP compared to nonsmokers. It can indicate that AOPP in the blood of AP patients is formed mainly as a result of free radical production induced in the course of disease, but tobacco smoke exposure can be considered as a factor exacerbating this process. The process of albumin oxidation to AOPP occurred in the course of AP in both smokers and nonsmokers; wherein in smokers, it is more intensive. It was confirmed that a strong positive correlation between cotinine and AOPP concentrations was observed in the group of smoking AP patients. Albumin oxidation causes a Zn release from this protein as a major antioxidative factor, which intensifies oxidative stress. Additionally, the association of cotinine and IL-6 concentrations or the concentrations of IL-6 and ET-1 that was found in the group of smoking AP patients can indicate that oxidative stress induced by tobacco smoke amplifies the inflammatory cascade and it can be an important factor for the pathogenesis of acute pancreatitis, which in other studies were also shown [32, 34, 35]. Proinflammatory effect of tobacco smoke xenobiotics in AP patients can also indicate an increase in both AAT and AGP concentrations causing, furthermore, a decrease in albumin concentration, which confirmed a positive correlation of these proteins. Hepatic mRNA upregulation of those acute-phase proteins, especially AGP, is associated with a decrease in albumin synthesis, and the severity of disease as in other studies was shown [13, 15, 36]. Therefore, a decrease in albumin concentration accompanied by a gradually increasing AOPP/albumin ratio in the blood of smoking AP patients can explain a greater number of AP attacks in this group compared to nonsmokers, as shown in Table 1.

An increase in ROS production modulating stress-signalling pathway and increasing the synthesis of proinflammatory molecules can cause a potential tissue damage of key target organs, such as the vasculature and pancreas [31]. Therefore, in our study, the changes in the concentration of resistin and ET-1 as markers of vascular endothelium damage were estimated. In other studies, it was shown that an increase in IL-6 concentration can induce the resistin expression in peripheral blood mononuclear cells indicating the role of this hormone in the inflammatory process [37–41]. Resistin concentration positively correlated with inflammatory markers, the pathological scores of the pancreatic tissues [42, 43] and organ failure in patients with AP [44]. It was noted that the concentration of this peptide was significantly higher in AP patients than controls [42, 45, 46]. These results are consistent with our study, in which it was demonstrated that the resistin concentration in the blood of AP patients increased 2-fold compared to control groups, in both smokers and nonsmokers. Additionally, we observed that resistin concentration was successfully enhanced during hospitalization in the blood of nonsmoking patients with AP, which in other studies was also shown [42, 45, 46]. However, no significant changes in resistin level during hospitalization of smoking AP patients can be associated with the effect of tobacco smoke components, mainly nicotine, that can cause lipid catabolism and decrease in abdominal fat and body weight [47]. It can cause a decrease in expression of resistin by reduction of tissue availability responsible for its production. On the other hand, in our study, an influence of tobacco smoke exposure on increased resistin concentration was demonstrated, as evidenced by a positive correlation of resistin concentration and Cd level in the blood of smoking AP patients.

Increased resistin concentration can act as a mediator of endothelial dysfunction by promotion of endothelial cell activation via the release of ET-1 [39, 48–50]. In our studies, a positive correlation of ET-1 and resistin showed in the group of smoking patients with AP can confirm that an increase in resistin concentration escalated ET-1 secretion from endothelial cells. Simultaneously, it can indicate that the changes in resistin concentration in AP patients appear earlier than in ET-1 concentration, in which no changes in AP patients during hospitalization were observed. It can indicate that resistin can be a predictor of vascular endothelium damage causing the release of ET-1 as marker of endothelial dysfunction. The lack of changes in ET-1 concentration between the examined group can be also an effect of protective action of increased AGP concentration on the endothelium, as other studies have mentioned [13] and confirmed by the negative correlation of these proteins in the group of healthy smokers in our study. On the other hand, in the presented study, we examined the patients with mild AP and not complicated the course of disease. This fact can have an influence on blood ET-1 concentration, because in previous studies [17] it was shown that elevated ET-1 concentration is a marker of the progress of the disease and the efficacy of the treatment. In our study, we confirmed this thesis, because during tobacco smoke exposure, which is considered as a major factor of AP, a higher range of ET-1 concentration in comparison to nonsmokers was noted.

**Figure 1:**
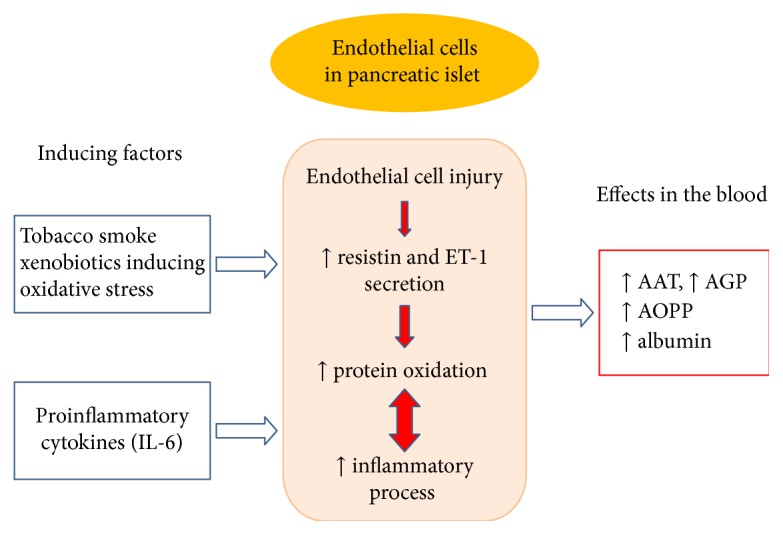
Participation of tobacco smoke xenobiotics in the intensification of oxidative stress and inflammatory process in the way of endothelial cell damage and resistin secretion.

Other studies have shown that an increased resistin and ET-1 concentration may in return promote oxidative stress in the vessel wall [51, 52], which in the presented study was confirmed by positive correlations of ET-1 or resistin concentrations with the value of AOPP/albumin ratio in the group of smoking patients with AP. Additionally, it was shown that an increase in resistin concentration was accompanied by a decrease in albumin concentration in this group, which was also confirmed by the negative association of these parameters. These results can indicate that intensive production of free radicals during AP caused oxidation of albumin to AOPP mediated by elevated resistin concentration. It was evidenced that resistin can have prooxidative properties and can act as prooxidant mediator for endothelial dysfunction, which in other studies was reported [19]. An increase in resistin concentration as a prooxidant in the blood of patients with AP can also induce the production of acute-phase proteins, which was reflected in the correlation of these parameters. It can indicate the involvement of AGP in the reduction of smoke-induced oxidative stress in healthy smokers and confirmed that AGP and AAT may be also produced as the response to oxidative damage induced by resistin action in the course of AP. This process in Figure 1() was presented.

Taking together, oxidative stress induced during tobacco smoke exposure and inflammation can be related to each other in the way of the activation of proinflammatory mediators. It can lead to intensification of inflammatory response reflected by elevated acute phase protein concentration. The progress in inflammation in return can induce free radical production, which was manifested in a decrease in blood albumin concentration accompanied by increased AOPP concentration. Consequently, it can cause endothelium damage and an increase in resistin concentration, which can be involved in the disturbances in the circulatory systems and lead to pancreatic hypoxia. It can contribute to the progression of acute pancreatitis.

## 5. Conclusions

1. An increase in IL-6 concentration in healthy subjects and the correlation of IL-6 with cotinine level in the blood of patients with AP confirmed the proinflammatory effect of tobacco smoke exposure.
2. A decrease in albumin concentration as an important blood antioxidant in AP patients indicates the involvement of this protein in inflammatory process.
3. A 2-fold increase in AOPP/albumin ratio in the blood of patients with AP compared to healthy subjects is an effect of intensified albumin oxidation to AOPP as a result of involvement of this protein in oxidative stress neutralization during AP.
4. An increased resistin concentration in the blood of AP patients negatively correlating with albumin concentration can have a prooxidative effect resulting in enhanced level of oxidative stress markers, especially in AOPP concentration.
5. An increased resistin concentration can intensify the production of acute phase proteins (AAT and AGP) in the course of AP.
6. Resistin can be a predictor of vascular endothelium damage causing the release of ET-1 as a marker of endothelial dysfunction.

## Figures and Tables

**Table 1:** Criteria for the inclusion of patients to the study.

Criteria	Circumstances for the inclusion
Clinical symptoms, personal interview	(i) Acute onset of a persistent
	(ii) Severe, epigastric pain with tenderness on palpation on physical examination

Laboratory tests	Serum amylase or lipase levels elevated to three times or greater than the upper limit of normal

Imaging	Characteristic findings of acute pancreatitis on contrast-enhanced computed tomography (CT), magnetic resonance imaging (MRI), or transabdominal ultrasonography
	(i) The patients with abdominal pain that were not characteristic for acute pancreatitis or serum amylase or lipase levels that were less than three times the upper limit of normal, or in whom the diagnosis was uncertain—abdominal imaging with a contrast-enhanced abdominal CT scan to establish the diagnosis of acute pancreatitis and to exclude other causes of acute abdominal pain
	(ii) The patients with severe contrast allergy or renal failure—abdominal MRI without gadolinium

Intravenous fluid	Approximately 4 liters of crystalloid solutions under the control of RR/HR, hematocrit, and hourly diuresis, which were modified relative to the dose of intravenous fluids (5–10 ml/kg/h) and a degree of hydration considering the signs fluid overload and edema

Treatment	(i) Any preventive antibiotics
	(ii) Oral low-fat diet
	(iii) Analgesics (if necessary)

**Table 2:** Demographic and clinical characteristics of the study population.

Parameters	Control group		Patients with AP	
	Nonsmokers Median (range)	Smokers Median (range)	Nonsmokers Median (range)	Smokers Median (range)
Age (years)	22 (20–46)	23 (20–31)	53^(1)^ (26–84)	44^(2)^ (29–76)
BMI (kg/m^2^)	21.60 (17.63–29.72)	21.92 (17.58–28.31)	25.33^(1)^ (21.60–38.09)	22.86 (17.11–34.53)
Ranson criteria (score)	NA	NA	2.00 (2.00–4.00)	2.00 (1.00–4.00)
The number of AP attacks in the past	NA	NA	1–3	2–10
Pack years of smoking	NA	3.00 (0.50–12.00)	NA	15.00 (1.00–61.50)
Alcohol abusers (%)	NA	NA	14%	67%
Alcohol exposure (g of ethanol/day)	NA	NA	14.30	110.00 (12.50–325.00)

NA: not applicable. ^(1)^*p* < 0.05 compared to nonsmokers of control group. ^(2)^*p* < 0.05 compared to smokers of control group.

**Table 3:** Cotinine, Cd, IL-6, AAT, and AGP concentrations in the blood of healthy subjects and patients with AP in the 1st, 3rd, and 7th day of hospitalization.

Parameters	Control group		Patients with AP	
	Nonsmokers Median (range)	Smokers Median (range)	Nonsmokers Median (range)	Smokers Median (range)
Cotinine (ng/ml)				
1st day	0.98 (0.02–14.51)	72.62^(1)^ (24.48–215.97)	0.75 (0.11–2.53)	130.22^(2), (3)^ (71.37–245.11)
3rd day			0.35 (0.09–1.24)	97.69^(2)^ (2.16–246.48)
7th day			0.22 (0.05–0.91)	104.93^(2), (4)^ (0.20–174.68)
Cd (*μ*g/l)				
1st day	0.12 (0.0–0.48)	0.91^(1)^ (0.22–1.37)	0.79^(1)^ (0.14–2.69)	4.17^(2), (3)^ (0.83–12.48)
3rd day			0.91 (0.38–4.97)	4.20^(2)^ (0.95–19.37)
7th day			1.09 (0.31–2.83)	3.74^(2)^ (0.31–8.83)
IL-6 (pg/ml)				
1st day	0.26 (0.10–14.63)	0.44^(1)^ (0.10–98.40)	3.20^(1)^ (2.09–31.93)	2.02^(3)^ (1.52–102.06)
3rd day			4.05 (2.59–24.47)	2.45 (1.29–75.62)
7th day			4.05 (2.48–6.93)	3.62 (1.32–106.58)
AAT (g/l)				
1st day	1.69 (1.09–2.16)	1.86 (0.72–3.31)	2.60^(1)^ (1.08–4.39)	2.04 (0.62–3.79)
3rd day			2.05 (1.13–3.05)	2.36 (1.01–5.02)
7th day			4.57^(4), (5)^ (2.17–7.75)	3.74^(4), (5)^ (2.83–7.63)
AGP (g/l)				
1st day	1.20 (0.85–1.52)	1.31 (0.79–1.87)	2.22^(1)^ (1.20–4.76)	2.93^(3)^ (1.92–4.16)
3rd day			2.94 (1.92–4.31)	2.82 (1.72–5.17)
7th day			3.53 (1.14–7.15)	4.65^(4), (5)^ (2.23–8.87)

^(1)^ *p* < 0.05 compared to nonsmokers of control group. ^(2)^*p* < 0.05 compared to nonsmoking patients with AP. ^(3)^*p* < 0.05 compared to smokers of control group. ^(4)^*p* < 0.05 compared to the 1st day of hospitalization. ^(5)^*p* < 0.05 compared to the 3rd day of hospitalization.

**Table 4:** The concentrations of albumin, AOPP, the values of AOPP/albumin ratio, resistin, and ET-1 concentrations in the blood of healthy subjects and patients with AP in the 1st, 3rd, and 7th day of hospitalization.

Parameters	Control group		Patients with AP	
	Nonsmokers Median (range)	Smokers Median (range)	Nonsmokers Median (range)	Smokers Median (range)
Albumin (g/l)				
1st day	68.02 (53.77–85.47)	67.36 (47.92–86.04)	46.79^(1)^ (33.21–57.17)	48.30^(2)^ (29.06–61.51)
3rd day			42.45 (28.30–58.30)	38.96^(3)^ (29.43–52.26)
7th day			44.53 (27.92–51.13)	37.92^(3)^ (28.87–53.77)
AOPP (*μ*mol/l)				
1st day	32.15 (8.47–62.25)	27.58 (9.38–62.25)	43.18^(1)^ (20.14–73.64)	50.26^(2)^ (14.19–85.25)
3rd day			46.84 (28.74-75.91)	56.77 (29.04–73.59)
7th day			58.76 (35.20–76.11)	65.25^(3), (4)^ (45.71–100.51)
AOPP/albumin (*μ*mol/g)				
1st day	0.48 (0.12–0.96)	0.41 (0.01–1.21)	0.97^(1)^ (0.40–2.21)	1.11^(2)^ (0.31–2.04)
3rd day			0.89 (0.74–2.68)	1.42^(3)^ (0.84–2.32)
7th day			1.32 (0.79–2.73)	1.56^(3)^ (0.93–2.88)
Resistin (ng/ml)				
1st day	11.62 (5.11–22.04)	9.98 (8.43–14.87)	22.48^(1)^ (16.09–27.60)	18.17^(2)^ (11.75–45.43)
3rd day			34.06 (8.95–56.53)	20.75 (9.62–70.69)
7th day			47.55^(3)^ (9.91–74.75)	25.60 (14.81–75.12)
ET-1 (pg/ml)				
1st day	1.52 (0.67–2.49)	1.21 (0.81–4.40)	1.22 (0.32–2.11)	0.97 (0.39–3.68)
3rd day			1.23 (1.01–1.38)	1.63 (1.07–2.20)
7th day			1.48 (1.23–1.91)	1.67 (1.44–1.90)

^(1)^ ^(2)^ ^(3)^ ^(4)^ *p* < 0.05 compared to nonsmokers of control group. *p* < 0.05 compared to smokers of control group. *p* < 0.05 compared to the 1st day of hospitalization. *p* < 0.05 compared to the 3rd day of hospitalization.

**Table 5:** The correlation coefficients resulted for linear regression performed between parameters determined in the blood of healthy subjects and patients with AP.

Correlations	*r*	*i*
*Smoking control group*		
AAT concentration–AGP concentration	0.5819	0.0471
AOPP concentration–albumin concentration	−0.6075	0.0126
AGP concentration–resistin concentration	**0.9554**	0.0112
AGP concentration–ET-1 concentration	**−0.8134**	0.0077
*Nonsmoking patients with AP*		
AAT concentration–AGP concentration	**0.8685**	0.0112
AGP concentration–albumin concentration	**−0.7438**	0.0344
AAT concentration–AOPP/albumin ratio	**0.8155**	0.0136
AAT concentration–resistin concentration	**0.9338**	0.0202
AGP concentration–resistin concentration	**0.9693**	0.0064
*Smoking patients with AP*		
Cotinine concentration–IL-6 concentration	0.5315	0.0232
Cotinine concentration–AOPP concentration	0.6467	0.0050
AAT concentration–albumin concentration	−0.5611	0.0237
AGP concentration–Cd concentration	**0.6906**	0.0044
Resistin concentration–Cd concentration	0.5649	0.0353
Resistin concentration–albumin concentration	**−0.6546**	0.0059
Resistin concentration–AOPP/albumin ratio	**0.6045**	0.0131
Resistin concentration–ET-1 concentration	**0.6466**	0.0131
ET-1 concentration–IL-6 concentration	**0.7036**	0.0024
ET-1 concentration–AOPP/albumin ratio	0.5202	0.0389
